# HIV Diagnosis and Treatment through Advanced Technologies

**DOI:** 10.3389/fpubh.2017.00032

**Published:** 2017-03-07

**Authors:** Hafiza Fizzah Zulfiqar, Aneeqa Javed, Sumbal, Bakht Afroze, Qurban Ali, Khadija Akbar, Tariq Nadeem, Muhammad Adeel Rana, Zaheer Ahmad Nazar, Idrees Ahmad Nasir, Tayyab Husnain

**Affiliations:** ^1^Centre of Excellence in Molecular Biology, University of the Punjab, Lahore, Pakistan; ^2^Department of Microbiology, Quaid-i-Azam University, Islamabad, Pakistan

**Keywords:** HIV, retoviridae, lentivirus, macrophages, antiretroviral therapy, CRISPR/Cas9

## Abstract

Human immunodeficiency virus (HIV) is the chief contributor to global burden of disease. In 2010, HIV was the fifth leading cause of disability-adjusted life years in people of all ages and leading cause for people aged 30–44 years. It is classified as a member of the family Retroviridae and genus Lentivirus based on the biological, morphological, and genetic properties. It infects different cells of the immune system, such as CD4+ T cells (T-helper cells), dendritic cells, and macrophages. HIV has two subtypes: HIV-1 and HIV-2. Among these strains, HIV-1 is the most virulent and pathogenic. Advanced diagnostic methods are exploring new ways of treatment and contributing in the reduction of HIV cases. The diagnostic techniques like PCR, rapid test, EIA, p24 antigen, and western blot have markedly upgraded the diagnosis of HIV. Antiretroviral therapy and vaccines are promising candidates in providing therapeutic and preventive regimes, respectively. Invention of CRISPR/Cas9 is a breakthrough in the field of HIV disease management.

## Background

Human immunodeficiency virus (HIV) originates from a monkey infecting virus, simian immunodeficiency virus (SIV), and a number of theories have been described in this regard. Many of the epidemiological, phylogenetic, and genomic characteristics of HIV are similar to those of SIV, and this strongly supports the idea of cross species transmission (1). HIV is classified as a member of the family Retroviridae and genus Lentivirus based on the biological, morphological, and genetic properties (2). Initial cases of HIV were reported in 1981 to Centre for Disease Control, and the virus was first isolated from patients with severe immune deficiency, later termed as Acquired Immune Deficiency Syndrome (AIDS), in 1983 (3). Since then, virology of HIV and pathogenesis of infection are constantly being studied. Two types of HIV have been isolated and characterized from patients infected with the virus: HIV-1 and HIV-2. Among these strains, HIV-1 is the most virulent and pathogenic, and when people normally talk about HIV without stating the type of virus they are referring to HIV-1 (4). On the other hand, HIV-2 is only constrained to some areas of Central and Western Africa (2). A detailed investigation on HIV structure and its mechanism of infection have not only allowed to characterize and develop new and effective vaccines and drugs but has also described new approaches for diagnosis of HIV at the laboratory scale (2).

## Epidemiology

Human immunodeficiency virus is the chief contributor to global burden of disease. In 2010, HIV was the fifth leading cause of disability-adjusted life years in people of all ages, and leading cause for people aged 30–44 years. In 2005, AIDS-related deaths peaked to 2.3 million globally, but reduced to 1.6 million by 2012 (5). Group M of HIV-1 is the major cause of worldwide HIV epidemic. There are nine known phylogenetic subtypes, sub-subtypes of Group M, clades (A–K) and an inter-subtype circulating recombinant forms (CRFs) among which inter-subtype genetic diversity is 25% for the *env* gene and 15% for the *gag* gene. Subtypes and sub-subtypes are a result of founder effects at various time periods in the past, whereas if two different subtypes co-infect a patient it gives rise to the inter-subtype recombinants. These recombinants are called CRFs if they have a significant epidemic spread. Subtype B of HIV-1 dominates in Australia, Americas, and Europe, whereas subtype C predominates in India and Africa (which accounted for 48% of all the HIV-1 cases in 2007). In 2012, approximately 35.3 million individuals were living with HIV, with the highest global burden of HIV (70.8%) in Sub-saharan Africa (6). However, increasing access to antiretroviral therapies has significantly improved the global epidemiology of HIV infection. There has not been a significant increase in the prevalence of HIV globally, with 31 million cases reported in 2002 to 35.3 million cases reported in 2012. This is largely because people on antiretroviral therapies are living longer than before, while the global incidence has reduced by approximately 1 million from 2002 to 2012 (7). According to the survey report of UNAIDS (2015), globally about 36.7 million people suffered from HIV infection and among them approximately 2.1 million new HIV infections were reported (8).

## HIV Tropism

Human immunodeficiency virus infects different cells of the immune system, such as CD4+ T cells (T-helper cells), dendritic cells, and macrophages. However, CD4+ T lymphocytes are the main target of HIV, and after infection, these cells are used by HIV as host to make copies and infect other cells of the body (7). This leads to collapse of the immune system as the number of CD4+ cells in the body decrease. This decline in the number of CD4+ cells indicates the development of HIV to AIDS. Mostly CCR5 and CXCR4 chemokine receptors are commonly used by these viruses for gaining entry into the T-helper cells. However, in some cells, such as astrocytes and renal epithelial cells, CD4-independent HIV infection occurs and subsequent pathogenesis is dependent upon HIVV gene expression. Virus replication is restricted or promoted in specific cell types by the interaction of several host proteins with proteins or DNA of HIV (4, 7, 9).

## HIV Structure and Genome Organization

Mature HIV virions are 100–120 nm in diameter spherical structures consisting of a lipid bilayer membrane which encloses a dense truncated cone-shaped nucleocapsid (core). The core contains two 9.8-kb long positive sense, single stranded, linear RNA molecules, molecules to initiate cDNA synthesis, cellular tRNA, Gag polyprotein, viral envelope (Env) protein and three enzymes: reverse transcriptase (RT), viral protease (PR), integrase (IN), and some other cellular factors (10, 11). The HIV genome contains accessory and regulatory genes flanked by long terminal repeats (LTR). The viral genome has a total of nine genes which can be divided into three functional groups: structural genes, *Gag, Pol*, and *Env*; regulatory genes, *Tat* and *Rev*; accessory genes, *Vpu, Vpr, Vif*, and *Nef* (3).

The *Gag* gene codes for the core protein, *pol* gene codes for RT, protease, integrase, and *Env* gene codes for the Envelope protein (gp160). The Tat and Rev regulatory proteins function as RNA-binding proteins. In addition to RNA binding, Tat proteins also act as activators of transcription ensuring that full length genomes of HIV are formed. Rev protein also helps in shift of gene expression of HIV from early to late phase (3). On the other hand, accessory proteins are multifunctional. *Nef* or negative factor is involved in T-cell activation, down regulation of existing major histocompatibility complex (MHC) I, and CD4 on the cell surface by degranulation in lysosomes and also stimulate virion infectivity. *Vpr* acts as a nucleo-cytoplasmic transport factor which permits HIV to infect non-dividing cells. *Vpu* enhances release of virion through the development of an ion channel and also down-modulates expression of CD4 through ubiquitin-mediated degradation. Replication of HIV in lymphocytes, monocytes, and macrophages is regulated by *Vif* (3). The envelope of the virion contains the transmembrane proteins, gp120 and gp41, which project outwards from the virion in the form of spikes (up to 72 in number). Being a highly immunogenic protein, gp120, which binds to the CD4 receptor, is a suitable target for majority of host antibodies. Most of these strain-specific antibodies block the interaction of CD4 receptors with gp120 protein by binding to these receptors. The matrix lying underneath the lipid bilayer consists of Gag protein 17 (viral gag protein cleavage product). The core or capsid contains a covering of p24 protein (product of gag gene), and a third Gag protein p7 (1).

## HIV Life Cycle

Human immunodeficiency virus viral entry steps are divided into basically three steps: (1) binding, (2) activation, and (3) fusion. Major HIV-1 and HIV-2 receptors and co-receptors are CD4 and CCR5, CXCR4, respectively. The cycle starts with the recognition of HIV-enveloped trimeric complex, gp120 and gp41 with CD4 receptor (58 kDa monomeric glycoprotein) major co-receptor of MHC class II molecule, on cell surface (2, 10). Upon binding of CD4 with gp120, a conformational change occurs resulting in exposure of gp120 domain where CCR5 chemokine co-receptors bind. So far, 17 chemokine receptor ligands are identified in this process (2). Following double binding of gp120, a stable attachment complex formed which allows the N-terminal side of gp120 peptide penetration in plasma membrane. In gp41 protein, HR1 and HR2 sequences act together and form a hairpin structure of gp41, which causes fusion of viral and cellular membrane (10). After fusion viral core is released in cytoplasm, uncoating of viral capsid occurs mediated by MA, Nef, and Vif protein factors of virus (1). By viral RT ribonuclease H site viral RNA is transcribed into DNA starting from primer binding site. After completion of transcription, ribonuclease H breaks the dsRNA\DNA hybrid and by RT polymerization active site converted into dsDNA. POBE3G protein presence means a lot with reference to reverse transcription fidelity (2).

Proviral status is obtained by integration of this dsDNA into host cell genome by integrase enzyme. The integrase protein produces sticky ends at 3′ end of each DNA strand. Now modified viral DNA is exported to nucleus through nuclear pore, directed by viral Vpr, and integration function is accomplished by this integrase (10). For the viral genome to be expressed, the host genome integration site should be in active state (2, 12). Now, in the provirus state, the viral DNA may remain for several years in host genome and upon receiving activation signal expressed mRNA using host polymerase enzyme (4) Latently infected T cells, macrophages, monocytes, and microglial cells are major reservoirs of HIV genome. In active cell state, transcription of HIV genome starts due to host RNA polymerase II and other transcription factors by binding with viral LTRs. Following transcription, translation resulted in basal amount of proteins (Tat, Rev, and Nef). On adequate production of Tat, further transcription is controlled by binding of Tat with TAR elements on LTRs and other transcriptional cellular activators (10).

In early stages, multiply spliced mRNA produces Rev, Tat, and Nef. On achieving adequate amount of Rev, non-spliced and longer mRNAs are produced referred to polysome, resulting in the production of other viral proteins and genomic RNA. On the unspliced RNA RRE, Rev Response elements are present where Rev binds and causes the safe transportation, without splicing, to cell cytoplasm for translation (1). REV also causes expression of enzymatic and structural proteins and regulatory proteins inhibition so play role in producing mature virion. In cytoplasm, ENV gene is translated into gp160 glycosylated in ER resulted into mature gp120 and gp140 by HIV-1 protease (2). During translation, ribosome-1 frame shift resulted in gag pol proteins includes PR, RT, and IN. Nucleus of mature virions are formed by gag and pol gene proteins. From large 160 kDa precursor gag and pol proteins are formed cleaved by viral proteases into p24, p9, p7, p17 gag final products and pol products. This cleavage is necessary for infectious viral particles ENV proteins, which after translation move toward membrane and gets insert into it. Gag and gag–pol polyprotein also move toward cell membrane and started to assemble mediated by Gag polyprotein. Full size genomic RNA, cellular tRNAlys3primer, enzymes, and all cellular compounds become linked with immature viral core (10). Budding of immature virus takes place through plasma membrane. It is necessary to have less number of CD4 molecules on cell surface when virus assembly and budding occurs. Nef, ENV, and Vpu are involved in this process. Nef in early stages mediate the endocytosis and mortification of MHC class I and II molecules. In later stages, Npu induces the degradation of CD4 molecules. During budding, activation of protein protease takes place which auto-catalytically cleaves Gag and Gag–pol polyprotein resulted in structural proteins and viral enzymes. Further interactions of individual proteins with capsid, nucleocapsid protein resulted in conic nucleocapsid, and MA remain associated to viral envelop (10).

## Role of Human Leukocyte Antigen (HLA) and Killer Immunoglobulin-Like Receptor (KIR) Polymorphism in HIV Susceptibility and Disease Progression

Host–virus genetics and environmental factors play a major role in susceptibility and progression to disease, as well as treatment outcomes (12). The immune system of humans has evolved a wide range of methods to recognize and destroy various microbial pathogens (13). Genome-wide association studies (GWAS) have significantly demonstrated that HLA class I HLA-A, B, and C, encoded by genes within the human MHC on chromosome 6, play a key role in microbial recognition and host defense, and hence is the most important host genetic factor in HIV control (14). The main function of HLA-A, B, and C is the presentation of both host and foreign antigens on the surface of cells infested with viruses. This signals the Natural killer (NK) cells and antigen-specific CD8+ T cells of innate and adaptive immunity, respectively, which destroy the virus infected cells (13, 15). The regulation of NK cell activity is also determined by the interaction of HLA class I molecules with KIR, as HLA class I molecules act as ligands for KIR (16). Extreme diversity of KIR and HLA loci has a differential impact on HIV pathogenesis across individuals.

Human leukocyte antigens class I genes show extreme polymorphism, encoding thousands of alleles, with maximum variability shown in the peptide-binding groove. These allelic variants in the HLA molecules influence the affinity and specificity of peptide binding and recognition of antigens by cytotoxic T lymphocytes (CTLs) (17). Among the class I loci, HLA-B alleles are the most polymorphic and have a major influence on CD4+ T lymphocyte count, viral set point, and therefore, on the rate of progression to AIDS (18).

Genome-wide association studies have revealed that a single nucleotide polymorphism (SNP) in HCP5 gene, which is in linkage disequilibrium with HLA-B*5701 in Caucasians; an intronic variant in the HLA-B gene linked to HLA-B*5703 in African-Americans; and two variants 35 kb upstream in the HLA-C locus are among the most important allelic variants in HIV pathogenesis (14, 19). However, HLA-B*57 and its related alleles are of principal importance in terms of its effect on HIV, as it has a protective influence both in terms of viral load control and slow onset of the disease (20). Other HLA-B alleles that associate with HIV protection include HLA-B*58, HLA-B*51 HLA-B*27, whereas HLA-B*35 and HLA-B*53 are associated with increased susceptibility to HIV infection (13, 14, 17). Some HLA-B locus alleles are associated with poor CTL response, high viremia, and rapid progression to AIDS in Caucasians and African-American populations infected with HIV-1 subtype-B. HLA-B7 supertype is the most predominant in this regard (16, 18). Among the HLA-A alleles, A*23 and A*24 alleles are related with rapid disease progression, whereas HLA-A protective alleles include A25, A26, A68, A23, and A32 (17).

Another factor associated with an increase in infectious disease and AIDS progression is the HLA class I homozygosity at one or more loci, as this may decrease the number of viral epitopes that could be resented to CTLs (17). Moreover, individual showing more variation or heterozygosity, at the HLA locus have a selective advantage against infectious agent HIV and hence a delay in the onset of AIDS (18). This is attributed to the increased diversity of viral peptides presented to T lymphocytes in these patients (12, 16). Another study reveals that a functional polymorphism that results in the deletion of a chemokine receptor CCR5, a key cofactor, is associated with protection from HIV infection in homozygotes and slower progression of the disease in heterozygotes (21).

Human leukocyte antigens-B alleles exhibit two major types of motifs, Bw4 and Bw6. Flores-Villanueva and colleagues demonstrated that Bw4 homozygosity was associated with protection from HIV-1 viremia and AIDS (22). These Bw4 motifs also functions as KIR ligands, which can be used to interpret the role of NK cells in restricting viral replication, thus delaying the onset of the disease in HIV seroconverters and long-term non-progressors (16, 17). Strong synergistic relationship between HLA class I and KIR loci play a major role in regulating the activity of NK cells in HIV pathogenesis. Inhibitory and activating signals from KIR mediate the NK cell activity, and it has been found that an activating KIR allotype, KIR3DS1 in combination with HLA-Bw4 cluster show an epistatic interaction and is associated with delayed progression to disease (23). Moreover, HLA-B allele is not protective if KIR3DS1 is absent, and conversely, KIR3DS1 is related to rapid development of AIDS if particular HLA-B alleles are absent. Thus, it has been observed that the HLA-B/KIR genotype results in enhanced activation of NK cells, helps to contain viral load in early stages of infection and later by reducing the risk of opportunistic infections (but not HIV-related malignancies) (21).

## SNP Associated with HIV

After 25 years of efforts to combat the deadly effects of AIDS, no definitive cure or vaccine is available. Innovative drug and vaccine discovery needs a lot more understanding of all genetic factors especially those which may impact viral entry (24).

Many diseases (Infectious and inflammatory) have often shown strong association with MHC (genetic MHC); though, the basis for these associations remain evasive (25, 26). Many studies have suggested that several genetic factors have association with virus (HIV) infection progression, spontaneous and shared control of HIV and HCV viral load as well as the overrepresentation of many HLA antigens, such as HLA-B57 (26, 27).

CCR5Δ32 deletion mutation is the most highly studied genetic polymorphism being associated with delayed HIV-1 disease progression. Being extensively studied mutation, it has contributed in the development of CCR5 inhibitors [a new class of antiretroviral therapy (ART)] (28–30). Other genetic factors causes delayed HIV disease progression includes HLA-B27 (31, 32) and HLA-B57 (33). Among five non-synonymous SNPs in CD4 receptor (HIV-1 primary receptor), C868T (rs28919570) SNP is a substitutional change from amino acid tryptophan to arginine in the third domain of CD4 (34). According to another study conducted in a cohort of postpartum women in Kenya, it is concluded that mothers with the 868T allele show no association with increased HIV-1 disease progression (35). However, when balanced for baseline CD4 868T allele showed a delayed HIV-1 disease progression. To understand this protective mechanism, extensive studies are necessary in this regard (36).

Another SNP near *IL28B* gene codes for interferon λ3 (IFN-λ3) found to be associated with the spontaneous HCV clearance (37) and sustained virological response after HCV therapy (38). In previous genome-wide studies, IL28B SNP association with HIV control has not been discussed. The reason behind its strong linkage disequilibrium with other statistically analyzed SNPs shows the loss of association with viral control (27). According to previous research, protective alleles are not linked with spontaneous HIV control in African-American individuals but recently, a study suggested that *IL28B* CC genotype is independently linked with spontaneous HIV control in American white individuals (39, 40). The association between the *IL28B* rs12979860 SNP and spontaneous HIV control could be explained due to the antiviral activity of IFN-λ against HIV and other viruses. However, the proper mechanism of IL28B SNP influence on IFN-λ production and activity still needs further research (41). A number of studies suggested that innate immune system imparts a vital role in vulnerability to HIV infection and disease progression (42). So, taking 94 individuals with HIV/AIDS, 23 genes of innate immune system are sequenced using Ion Torrent next-generation sequencing platform. The resulted SNPs associated with viral load are reported in following genes; IL15RA (rs2229135; chr10:5995052), TLR7 (rs179008; chrX: 12903659), TRIM5 (rs11601507; chr11:5701074), KIR2DL1 (rs77397437; chr19:55286864), and KIR2DL3 (chr19:55251049). Two SNPs OAS2 (rs2072137; chr12:113440921) and OAS3 (chr12:113376388) are found to be linked with HIV infection progression (Table 1). Further confirmations of all these findings might be an important therapeutic target to understand the pathogenesis of the disease and for vaccine design (43).

**Table 1:** **Single-nucleotide polymorphisms (SNPs) associated with human immunodeficiency virus (HIV) infection and its statistical significance**.

Disease	SNP location	Strongest SNPs-risk allele	*P*-value	Reference
HIV-1 and acquired immune deficiency syndrome	Human leukocyte antigens (HLA)-C	rs9264942	5.9 × 10^−32^	(26, 43–50)
	HLA-B, HCP5	rs2395029	4.5 × 10^−35^	
	HLA-B	rs2523608	5.6 × 10^−10^	
	HLA-C	rs9264942	2.8 × 10^−35^	
	MICA	rs4418214	1.4 × 10^−34^	
	HLA-B, HCP5	rs2395029	9.7 × 10^−26^	
	PSORS1C3	rs3131018	4.2 × 10^−16^	
	HLA-B	rs2523608	8.9 × 10^−20^	
	Intergenic	rs2255221	3.5 × 10^−14^	
	HLA-B	rs2523590	1.7 × 10^−13^	
	Intergenic	rs9262632	1.0 × 10^−8^	
	ZNRD1, RNF39	rs9261174	1.8 × 10^−8^	
	PARD3B	rs11884476	3.4 × 10^−9^	
	HLA-B, HCP5	rs2395029	6.8 × 10^−10^	
	CXCR6	rs2234358	9.7 × 10^−10^	
	IL15RA chr10:5995052	rs2229135	–	
	TLR7 chrX: 12903659	rs179008	–	
	TRIM5 chr11:5701074	rs11601507	–	
	KIR2DL1 chr19:55286864	rs77397437	–	
	KIR2DL3 chr19:55251049	–	–	
	OAS2 chr12:113440921	rs2072137	–	
	OAS3	chr12:113376388		

## Diagnosis

After the inception of HIV in 1980s, its diagnostic tests have come into the way. Current techniques of HIV antibody detection are very sensitive as they can detect HIV antibody within or after 1 or 2 weeks of HIV infection. Additional confirmatory tests are also available for the accuracy of results. Many diagnostic laboratories have complex algorithm of testing for the accurate detection and precision of results (51–53). Early diagnosis of HIV saves a person in many aspects. First, it evades the further transmission of HIV and, second, makes the patient way in to ART. Due to updated recommendations and technological advances, HIV recognition and diagnosis became possible at early stages than in later stages. During late stages of infection mostly patients have AIDS-associated symptoms or immunodeficient with CD4 T cell count less than 200/μl (54, 55). HIV is diagnosed by many ways (Table 2) by the detection of antibodies in patient’s serum or plasma representing the presence of viral nucleic acid either by PCR chain reaction, p24 antigen, or rising viruses in cell culture. Most commonly antibody test is used for the detection of HIV infection. Seroconversion can be detected using high sensitive enzyme immunoassay within 2–3 weeks of infection in most of the cases. But for small number of cases, we are still in window period. P24 antigenic protein of virus may become visible or detectable before antibody detection.

**Table 2:** **Diagnostics tests for HIV infection**.

Type of test	Detection of DNA/RNA	Detection of antigen	Detection of antibody
PCR or viral load		–	–
P24 test	–		–
4th generation antigen/antibody (Ag/Ab) tests (p24+ ELISA, ELI, MEIA/ELFA/ECLIA): includes Architect, Duo, Combo/Combi, etc.	–		–
1st/2nd/3rd generation antigen only tests (ELISA, ELI, MEIA/ELFA/ECLIA)	–	–	
Rapid tests: finger prick and oral swab test are antibody only	–	–	
Western blot tests look for antibodies to specific HIV proteins	–	–	

Therefore, proper diagnosis depends upon the use of accurate testing by laboratories along with the availability of patient’s clinical history including high-risk symptoms associated with seroconversion illness (51). Reactive results can be confirmed by using alternative assay approaches. Fourth generation tests which are highly sensitive can be used for confirmation and detection of both p24 antigen and HIV antibodies. Many approaches are commercially available nowadays for HIV diagnosis and all are based on the same principle of antigen–antibody complexes (55). Positive EIA tests should be confirmed by another assay and the results must be repetitive for all laboratories (51). Based on ELISA many technological advances have been made. Highly automated and efficient modern tests are available which can generate results in less than 1 h. In such systems, viral antigen–antibody complexes are fixed with microparticles present on solid support. This method is named as microparticle enzyme immunoassay (55).

Western blot is another high sensitive immunoblot used as a confirmatory test. Radioimmunoprecepitation assay is used by some laboratories as a confirmatory test. In such an assay, viral radiolabel protein is complied with antibodies in the patient’s serum. Where antibody testing is insufficient, DNA PCR test is performed to check integration of viral genome in host’s genome. It is mostly performed in children of HIV+ve mother after 15 months of age.

It is also performed in patients having advance HIV infection but not appearing with its symptoms. Apart from all these, qualitative (viral load) and quantitative PCR are also available which aid in the beginning of drug therapy and its monitoring. Highly +ve screening tests which give unusual or intermediate Western blot results are then reconfirmed on specific HIV-2 blot (51).

### Rapid Tests

As the part of screening test, rapid test which gives reactive results are confirmed by Western blot assay. Rapid test is Quick and easy assay devoid of any complex equipment. For that reason, it is named as “point of care” test. Capillary blood in addition to plasma and serum can be used as a sample. In many other cases, urine or oral secretion can be used. Though, rapid tests are less sensitive than other types of test (56–59) but they give results within 15–30 min. Commonly, immune-chromatographic methods are the base of rapid test. Particle agglutination and immunefiltration techniques can be used as the bottom of rapid test (60, 61). Rapid tests today/these days are in use only to detect HIV antibodies not p24 viral antigen (third generation HIV test). Still primary HIV infection faces deficiencies in this regard. One-third of acute viral infection gives false negative results. Rapid test provides initial path to proceed next. Results are then confirmed with the standard HIV test in laboratory. In emergency situations, rapid tests are first priority to use. At the time of delivery in pregnant women rapid test proved to be a quicker method for the diagnosis of HIV infection. In countries having poor transportation, rapid test is considered as the best approach (55, 62).

### Diagnostic Window

The time period between the exposure to HIV and the point where biochemical measureable markers like antibodies, viral genome, and antigen (p24) are identified. HIV antibodies start producing within 2 weeks of virus transmission. Below pattern is observed in many cases.

**Table:** 

Cases (%)	Time period (in weeks)
60–65	After 4
80	After 6
90	After 8
95	After 12

P24 antigen is identified 5 days before seroconversion. So, fourth generation tests reduce the diagnostic gap by simultaneously detecting viral antibodies and p24 antigen. But, viral RNA is the first marker that can be detected 7 days before p24 antigen production. In most of the cases, HIV RNA can be detectable in the second week of viral exposure (55).

### EIA for HIV

Since 1980s, after the start of HIV testing, EIA became more sensitive and automated for HIV diagnosis. Fruitfully, it has shortened the window period but unfortunately it resulted in false positive results in most cases. So, it should be confirmed by various types of confirmatory tests usually Western blot. Laboratories may select first test with second EIA assay. Samples which give positive results in first assay and negative results in second assay are further confirmed by confirmatory tests in patients with high risk or symptoms (52).

### P24 Antigen

This test is based on EIA test and p24 antigen is detected using antibody. Positive results are further confirmed by neutralization test. Sometimes, p24 antigen is detected before antibodies detection in newly reported HIV infection in individuals. The usefulness of p24 antigen became clear by its use when patient is highly symptomatic, but EIA test is negative or positive but Western blot is positive. Follow-up antibody tests are further performed when patient’s p24 test is positive but antibody test is negative. Follow-up test will be positive within few weeks after first screening test in seroconverting patients. P24 antigen is not detectable in all seroconverting patients so, it is not unfailingly found in HIV antibody positive patients (51).

### Western Blot

Western Blot is a type of an immunoblot performed for the characterization of each viral protein. A nitrocellulose membrane/strip accommodates every viral protein arranged according to their molecular weight after performing polyacrylamide gel electrophoresis. Patient’s serum treated with this nitrocellulose strip produced a color band due to reaction of patient’s serum antibody with specific viral antigen. Color band is observed due to the presence antihuman alkaline phosphates labeled IgG conjugate and color developing solution. Colors are detected, and results are assessed by number any kinds of bands and manufacturer’s given guidelines. Patient’s sample must at least positively reacts with one core band and with one envelop band to be declared as positive. Samples having bands not according to positivity criteria are placed under indeterminate Western blot. So, further follow-up samples should be requested, commonly taken after 2–3 weeks of initial sample (51, 63, 64).

### Qualitative PCR

Amplification of viral nucleic acid to detect HIV infection is done by PCR (polymerase chain reaction). High sensitivity and specificity of PCR picks and choose a very small number of viral particles. Newborn babies from HIV-infected mother carry HIV antibodies up to 15 months of age. So, HIV antibody test is not reliable; therefore, detection should proceed through PCR. Taking into account Primers specificity, HIV result assessment should be declared cautiously as mother might be affected with HIV-2 or non B-HIV. PCR helps in solving indeterminate western blot results and detection of HIV in immunocompromised persons (51, 65).

### Quantitative RNA PCR and Genotyping

Before and after ART, RNA PCR is performed to monitor HIV-positive patients. In combination with CD4 count, RNA PCR is performed to decide when HIV therapy should start in clinical aspects. Genotyping is useful in assessing drug resistance in patients under the process of therapy and to assist physician what combination of drug therapy should be used. Quantitative PCR should not be taken as diagnostic test due to many false positive and negative results in such situation (51, 66, 67).

## Preventive HIV-1 Vaccine

### Induction of Neutralizing Antibodies

The main goal of early vaccine research was the development of HIV-1 vaccines that are capable of producing neutralizing antibodies. The efficiency and safety of different vaccines, such as gp120, gp160, parts of gp160, and peptides from gp160 were tested by conducting a lot of studies and research. These vaccines produced specific antibodies against HIV-1 strain *in vitro* but were unable to produce broadly neutralizing antibodies in HIV-1 variants derived from patients (68). In two Phase III trials two gp120-based vaccines were tested in healthy volunteers. Although the gp120 stimulated the production of antibodies but the rate of new infection did not decrease. These studies also indicate that the biological activity of the envelope molecule gp160 is difficult to be blocked by antibodies. The functionally important epitopes present in the grooves of the gp120 molecules, also covered by glycan shields and variable sequence loops, are not exposed before the binding of gp120 to the CD4 receptor. Therefore, the binding of gp120 to the CD4 molecule is difficult to be blocked by antibodies (69). A conformational change of the V3 loop occurs due to the binding of gp120 trimer to CD4 exposing a conserved high-affinity co-receptor binding site on the gp120 molecule, and the binding of co-receptor results in the fusion of the virus with the host cell membrane. To neutralize this process, antibodies against the V3 loop are required in high concentration. Despite this, the V3 loop–co-receptor interaction site is also protected by the gp120 trimer that prevents its neutralization by antibodies (70).

A number of factors contribute in limiting the production of broadly neutralizing antibodies (bNAbs), such as extensive Env glycosylation (71), the rapid evolution of HIV-1 Env proteins (72), conformational masking (69), and the scarcity of critical Env epitopes (69). Haynes and McMichael conducted a study in which they hypothesized that a robust humoral response against HIV is restricted due to the mimicry of neutralizing HIV-1 epitopes with host proteins. This phenomenon leads to the inactivation of responder B cells by immunological tolerance (73). Two human proteins, kynureninase (KYNU) and splicing factor 3b subunit 3 (SF3B3), were identified through two MPER-binding bNAbs (2F5 and 4E10), showing mimicry with the 2F5 and 4E10 HIV-1 epitopes (74). Experiments in knock-in mice, expressing the 2F5 or 4E10 V_H_DJ_H_ and V_L_J_L_ rearrangements, having compromised B-cell development indicate that generation of bNAbs to the 2F5 and 4E10 epitopes are blocked by immune tolerance. Moreover, different host epitopes were identified by bNAbs of HIV-1 (75–77). Human ubiquin-protein ligase 3A (UBE3A) is an example of these epitopes that was recognized by unrelated groups of CD4bs bNAbs. All these findings highlight that immunological tolerance instigated by host mimicry is also an important factor in limiting the humoral immunity against HIV-1 (78).

Among HIV-1-infected patients, only 30% produce neutralizing antibodies within 2–3 years after infection. The gp120 molecule shows high sequence variability by formation of rapid escape mutants leading to the escape of HIV-1 from antibodies so most of the patients develop antibodies which identify their own strain of HIV-1. Therefore, only small numbers of patients produce highly effective broadly cross-reacting neutralizing antibodies (bnAbs) (79).

Passive genetic immunization, that involves transfer of genes encoding highly active neutralizing antibodies or antibody-like immune-adhesins, offers a new approach that gained worldwide attention. Transfer of neutralizing antibodies genes through recombinant adeno-associated virus (AAV) vector in rhesus monkeys provided protection from HIV-1 infection, whereas protection could also be achieved by transfer of neutralizing antibodies genes through a new self-complementary AAV vector in a humanized mouse model (80). Recently, a study was conducted to examine the antiviral activity and safety of the 3BNC117 antibody in humans. This antibody is a potent CD4-binding site antibody cloned from a viremic controller. The mixture of 3BNC117 in approximately 30 mg/kg resulted in the reduction of viral load by 0.8–2.5 log copies/ml in HIV-1-infected viremic patients. Despite the development of resistance in few patients, the approach of passive antibody transfer might be suitable for the cure of HIV+ patients as well as for the prevention of mother-to-child transmission (81).

### Induction of HIV-1-Specific T Cells

The focus of vaccine development moves toward the vaccines that have the ability to stimulate HIV-1-specific T cell responses because of the difficulties faced in generation of neutralizing antibody responses. Cytotoxic T cells (CTL) are important in controlling the HIV-1 in humans and recognizing only the previously infected cells. Studies have demonstrated that HIV-1-specific CTLs have the potential to inhibit ongoing HIV-1 infection by suppressing the small foci of viral infection. But, if this vaccine fails to prevent infection of the host it still has the ability to decrease the level of viremia after infection (82). Viral setpoint is defined as the viral load of 4 months after infection. A vaccine that lowers the viral setpoint by half a log provides clinical benefit, because infectivity of the patients reduces with the reduction in the level of viremia (83). A good vaccine should comprise of highly conserved CTL epitopes for the individual HLA alleles in order to develop an effective CTL response. Through the formation of CTL escape mutants in T cell epitopes or in proteasome cleavage sites, HIV-1 escapes from the CTL recognition. Vaccines that insert viral peptides on HLA class I molecules of dendritic cells are capable of inducing the CTLs by displaying these peptides to CTLs. Live attenuated viruses and DNA vaccines are not promising due to different reasons in humans. Beside these, lipopeptides stimulate CTL production by displaying only few repertoires of epitopes (84).

### Recombinant Viral Vectors

The induction of CTLs can be achieved by using Recombinant vectors that avoid the problems associated with live attenuated viruses. The performance of different vectors, such as adenovirus 5 (Ad5) vectors, ALVAC canarypox viruses, adenovirus-associated virus, and fowlpox vectors, has been assessed through several clinical studies. A number of clinical trials have been carried out to test the efficacy of different vaccines (85).

A rhesus monkey cytomegalovirus vector containing recombinant SIV genes offers a different and new method. This vector plays an important role in generating permanent and broad CTL response by inducing unusual non-canonical CD8 T cells restricted by HLA-II antigens which cannot be downregulated by the viral nef protein. But, still, it is unclear whether such non-canonical CD8 T cells are present in humans or either their induction through vaccination is possible (86). Effective HIV-1 vaccines can be produced by therapeutic immunization of HIV-1-infected patients on ART following treatment interruption. During treatment interruption, the investigation of the vaccine’s ability to control HIV-1 replication provides an important way to determine the effectiveness of the vaccine in preventing the infection (85).

### RV 144: A Preventive Vaccine Trial

A number of effective treatments and preventive strategies have been established to tackle the severity of the illness caused by this deadly virus till now. However, all of these approaches are still not adequate to prevent the HIV transmission (87). The preventive vaccine against HIV still holds the position of a potential and promising candidate for the elimination of this disease from the globe (88). For the complete eradication of this pandemic up till now, researchers have conducted more than 250 phase I and phase II clinical trials (89). Initially neutralizing antibodies were considered as the potential candidates for designing vaccine against HIV, but the early vaccine trials based on the humoral immune response failed to stimulate the production of bNAbs (90). After this, the researchers directed theirs efforts toward developing a vaccine inducing cellular immune response in order to provide preventive vaccine against HIV (90).

An efficacy trial under the name of RV144 conducted in Thailand, created a new hope among scientists and researchers that a preventive vaccine against HIV is possible. The RV144 trial, the first effective vaccine trail, was based on a prime-boost concept involving upto 16,000 participants from 18–30 years old. The vaccinees were given four priming injections plus two boosting vaccine injections. The prime canarypox vaccine ALVAC-HIV (vCP1521) and boost gp120 subunit vaccine AIDSVAX B/E elicited both cellular and humoral immune responses. The vCP1521 was given at weeks 0 and 4 and vCP1521 together with AIDSVAX B/E given at weeks 12 and 24 (91). This combination vaccine surprisingly caused 31% reduction in HIV cases, providing minimal statistical significance (92). Primary analysis of correlates of risk of infection was conducted to check RV144 HIV-1 vaccine efficacy, which indicated that plasma IgG, specific to HIV-1 envEnv variable regions 1 and 2, have an indirect relation with infection risk while HIV-1 *Env*-specific plasma IgA responses are directly correlated with the rate of infection. However, secondary analyses suggested that the presence of low level of *Env*-specific IgA antibodies prevented the binding of ADCC-mediating mAb to HIV-1 *Env* glycoprotein 120, thus reducing the effects of protective antibodies (93). The findings of RV 144 trial further specified that in future these partially protective vaccines can be combined with other antiretroviral strategies in order to assess their synergistic effects (91, 94).

This trial opens up the door for more dedicated future research in the field of vaccine development and thus favoring new clinical trials (95). All the previous clinical trials on vaccine development which focused on the virus-enveloped proteins have utilized gp120 *Env* monomers or truncated gp160 (HVTN 505). The infectious virion bears trimers of gp160 having different antigenic properties and conformational epitopes form gp120 and truncated gp160 (HVTN 505) (96). In order to develop pathogenesis-driven approach, it is important to consider all these key difference to conduct more precise research in future (97).

## Treatment

Over the past 20 years, treatment of HIV has been markedly improved due to ART. Antiretroviral drugs are the valuable addition in the armamentarium of antiviral drugs (98). Few antiretroviral drugs were available in the preceding decade; however, in the mid 1990s, the development of various inhibitors for essential enzymes of HIV has revolutionized the HIV treatment (99). Initially, antiviral drugs were administered as monotherapy but later on the concept of combination therapy was introduced involving the administration of at least three drugs in combination. This treatment therapy was known as highly active antiretroviral therapy (HAART), and it has the potential to reduce the mortality and morbidity related to HIV-1 infection (100). This therapy plays a significant role in revision of immune system, by repressing viral replication and reducing the viral load below the level of detection (50 RNA copies/ml), which can be measured by elevated level of CD4+ T-lymphocyte. In 2010, European Union and United states guidelines suggested the use of HAART therapy when the levels of CD4 cells drop to 350/mm^3^ in blood, and this is the phase at which viral load exceeds to 10,000–100,000 copies per milliliter. Proper adherence to therapy results in repression of viral replication and increase life span of patients; however, it cannot eradicate HIV-1 infection. Drug–drug interactions, non-adherence, and reduced drug tolerability can impair the effects of HAART treatment (101).

Another milestone in the HIV treatment was achieved when scientists reported a case of 30-month-old Mississippian baby born with HIV infection. The infant received ART for the infection right after birth until HIV DNA and RNA levels met the standard diagnostic criteria. At the age of 18 months, the therapy was discontinued and baby was screened for the presence of viral load till the age of 30 months. Surprisingly through the age of 30 months the viral antibodies, DNA, and RNA levels remained undetectable in the plasma suggesting that chronic HIV infections can be cured in infants if provided with very early ART (100, 102).

### Types of Antiretroviral Agents

For the treatment of HIV infection more than 30 antiretroviral agents have been licensed till now (March 2015). These drugs are classified into the following different classes:

1. Nucleoside or nucleoside reverse transcriptase inhibitors (NRTIs);
2. Non-nucleoside reverse transcriptase inhibitors (NNRTIs);
3. Protease inhibitors (PIs);
4. Entry inhibitors (co-receptor antagonists and fusion inhibitors);
5. Integrase inhibitors (INSTIs).

For Therapeutic intervention now four targets are available in HIV life cycle: virus entry into cell and three enzymes (protease, RT, and integrase) (103).

#### Nucleoside Analogs (NRTIs)

The first class of drug which is sanctioned by FDA was NRTIs (99). Nucleoside reverse transcriptase inhibitors (NRTIs) which are also referred to as Nucleoside analogs (“nukes”) work by targeting RT enzyme of HIV. These inhibitors compete with normal cellular nucleosides by acting/performing as an alternative substrate (103). Deoxyribose sugar of these nucleoside analogs lacks hydroxyl group at 3′ position, which inhibits the formation of phosphodiester bond between incoming 5′ nucleoside triphosphates and NRTIs. This will lead to the termination of growing DNA chain of virus which can occur during DNA-dependent DNA synthesis or RNA-dependent DNA (99). These analogs are considered as pro-drugs as after endocytosis they use cellular kinases for phosphorylation in order to convert into active metabolite. Polyneuropathy, myelotoxicity, pancreatitis, and lactate acidosis, etc. are the long-term side effects caused by NRTIs (100). Eight FDA approved drugs are currently available which include; abacavir (ABC, Ziagen), zidovudine (AZT, Retrovir), didanosine (ddI, Videx), lamivudine (3TC, Epivir), emtricitabine (FTC, Emtriva), stavudine (d4T, Zerit), zalcitabine (ddC, Hivid), and Tenofovir disoprovil fumarate (TDF, Viread), a nucleotide RT 3′ inhibitor (104).

#### Non-Nucleoside Reverse Transcriptase Inhibitors

Non-nucleoside reverse transcriptase inhibitors target HIV RT enzyme along with nucleoside analog and were first described in 1990 (103). Proximal to active site, the formation of hydrophobic pocket and binding of NNRTIs to RT lead to the inhibition of HIV RT enzyme. Reduction in polymerase activity and spatial conformational changes in substrate-binding site take place upon binding of NNRTIs to RT enzyme (105). These NNRTIs are non-competitive inhibitors and are very specific in their action as they do not impede the RT of other lentiviruses (102).

The NNRTI pocket allows designing of extremely specific inhibitors with nominal side effects and reduced toxicities. In order to conduct enzymatic activity, it is not necessary for hydrophobic pocket to be conserved unlike active site or dNTP-binding site. Therefore, NNRTIs mutations are developed rapidly as compared to NRTIs mutations. Resistance mutation sites, non-overlapping inhibition mechanisms, and the fact that NNRTIs develops mutations rapidly emphasize clinicians to use this drug in combination with NRTIs (101, 106). NRTIs resistance is caused by two ways: discriminatory mutation and primer unblocking mutation. Discriminatory mutations are such type of mutations that allow the RT to distinguish between naturally occurring dNTPs and dideoxy-NRTI chain terminators. In this way, these mutations prevent the incorporation of NRTIs into the growing chain of viral DNA. K65R, L74V, M184V/I, Y115F, K70E/G, and the Q151M complex of mutations are the most common discriminatory mutations (107). While in primer unblocking mutations, phosphorylytic removal of NRTI triphosphate form the 3′ end of viral DNA take place. As these mutations are selected by zidovudine and stavudine, thymidine analog, therefore, these mutations are also known as thymidine analog mutations. K70R, L210W, K219Q/E, K219Q/E, T215Y/F, and D67N are common primer unblocking mutations (104). By interrupting the interaction between enzyme and inhibitor, NNRTI mutations impart resistance in three ways: by preventing the entry of inhibitor into binding pocket of NNRTIs (e.g., K103N), by affecting the interactions between residues and inhibitors lie along the binding pocket (e.g., Y181C), and by changing the size or conformation of binding pocket making it less specific for inhibitors (e.g., Y188L) (107). Efavirenz (EFV,Sustiva^®^), rilpivirine (RPV, Edurant^®^), delavirdine (DLV, Rescriptor^®^), etravirine (ETR, Intelence^®^), and nevirapine (NEV, Viramune^®^) are the FDA-approved drugs for the treatment of HIV infection (105).

#### Integrase Inhibitors

The recent enzyme of HIV-1 targeted successfully for drug development was integrase enzyme. In 2007, MK-0518 and Raltegravir was approved by FDA while Elvitegravir, GS-9137 are some inhibitors which are in clinical trials. These inhibitors distort the enzyme interaction with divalent cations (Mg^2+^) and correct positioning of viral DNA by binding near to the active site of enzyme (99). Retroviral enzyme integrase catalyzes a two-step reaction known as integration process. The process involves strand transfer and 3′ processing through coordination of divalent ions (Mn^2+^ or Mg^2+^) provided by three amino acids at locations E152, D64, and D116 (108). After reverse transcription, conserved dinucleotides at 3′end of double stranded DNA are cleaved by integrase. This will produce overhangs of dinucleotides on both ends of genome and a whole process is referred as 3′ processing reaction. While in strand transfer reaction, bounded integrase at 3′end of DNA translocate viral double stranded DNA into nucleus where it catalyzes the incorporation of viral DNA into host genome.

Although strand transfer reaction and 3′ processing is catalyzed by integrase enzyme but effective compounds for HIV treatment are considered to be those that can prevent the strand transfer reaction and are called integrase strand transfer inhibitors (INSTIs). They are considered as effective inhibitors as they bind to the enzymes already attached with viral DNA (102). Consequently, INSTIs are composed of two vital components: hydrophobic group and metal binding pharmacophore. The active site magnesium is sequestered by metal binding pharmacophore while hydrophobic group is responsible for interacting with enzyme and viral DNA in the complex (99). Mutations which impart resistance to INSTIs are nearly always present within the active site of integrase enzyme close to the triad of amino acids that helps to coordinate with vital magnesium cofactors. Detrimental effects on viral replication and enzymatic function are caused by these mutations (109).

#### Protease Inhibitors

Another class of inhibitors targets the protease enzyme of HIV-1 and is called as PIs. Protease enzymes catalyze the processing of gag–pol polyprotein precursor and virion gag and are essential for viral maturation (102). Ten PIs which have been sanctioned by FDA are: atazanavir (ATZ, Reyataz), darunavir (TMC114, Prezista), fosamprenavir (Lexiva), amprenavir (APV, Agenerase), indinavir (IDV, Crixivan), lopinavir (LPV), ritonavir (RTV, Norvir), saquinavir (SQV, Fortovase/Invirase), tipranavir (TPV, Aptivus), and nelfinavir (NFV, Viracept). Initially, it was anticipated that PIs will rarely develop mutations due to their small size and essential role in HIV-1 life cycle. But great plasticity was exhibited by protease gene as greater than 20 substitutions and polymorphism in 49 codons was observed. These substitutions and codon polymorphism were known to be related with resistance (99). Primary resistance mutation in most of PIs occurs adjacent to the active site of enzyme causing changes in amino acid, which has detrimental effects on viral replicative fitness. Besides mutation in protease gene, alterations in the eight important cleavage site also confer resistance to PIs (99, 110).

#### Entry Inhibitors

Binding of gp120 to the cell surface CD4 receptor initiates the entry of HIV-1 into the host cell. Within gp120, structural elements are present, which upon binding to CD4 expose and attach to one of the two co-receptors. After binding of structural elements of gp120 with co-receptor, the integration of transmembrane subunit gp41 into cell membrane takes place leading to fusion of virus and cell membrane. In order to inhibit the entry of HIV-1 into cell, a number of drugs have been developed which include: enfuvirtide (ENF), maraviroc (MVC), and ibalizumab (111, 112). On the basis of disruption of different steps in HIV-1 life cycle, entry inhibitors are further subdivided into different classes (99).

##### Fusion Inhibitors

Fusion of HIV-1 to host cell membrane is initiated by the interaction between two homologous domains in gp41, a viral protein. Fusion inhibitors, heterologous proteins, were designed to mimic one of these domains which upon binding cause disruption of intramolecular interaction of viral protein. Alpha-helical peptides provide substantial antiviral activity by mimicking to leucine zipper domain (99). Only sanctioned fusion inhibitor is Enfuvirtide, which prevents the interactions of gp41 hairpin formation. Folding of two complementary segments of gp41into one another cause shortening of protein thus bringing host and viral cell membranes together, and this process is termed as hair pin formation (102).

##### Attachment Inhibitors

Attachment inhibitors inhibit the interaction of gp120 with CD4 that block interaction of envelop with co-receptors (CXCR4 and CCR5) that ultimately stop the cellular and viral membrane fusion. These inhibitors are effective against CXCR4 and CCR5 tropic strain as compared to chemokine receptors. PRO542, TNX-355, and BMS48804 are FDA-approved attachment inhibitors which are currently available (113).

##### Small-Molecule CCR5 Antagonists

Another class of drug which can effectively inhibit the entry of HIV-1 into host cell is small-molecule antagonist of CCR5 co-receptor. The C–C chemokine receptor type 5 (CCR5), a 40.6-kDa protein having about 352 amino acid, belongs to a huge family of chemokine receptor (114). A variety of cell population including memory T cell, antigen presenting cells (macrophages and dendritic cell), fibroblast, astrocyte, vascular smooth muscle, etc., expresses these receptors to their cell surface (115–117).

The HIV virus uses CCR5 as a tool for entering into the cell alongside cell to cell spread making it definitely the significant co-receptor for HIV (118, 119). They work allosterically by preventing the attachment of gp120 of HIV-1 with host cell CCR5 (104). The binding of receptor antagonists with hydrophobic pocket of CCR5 causes a conformational change in the receptor making its recognition difficult by viral gp120. Three antagonists (VCV, MVC, and Aplaviroc) are currently available which have the potency to inhibit viral replication in humans (99). The role of CCR5 in HIV replication and its genetic polymorphism related to disease control and HIV resistance encourages the scientists to consider this key receptor for the treatment of HIV (120, 121).

The dramatic case of Berlin patient further highlighted the significance of CCR5 for the treatment of HIV. In 2007, Timothy Brown, lived in Berlin, suffered from HIV infection and later developed acute myeloid leukemia for which he went through a bone marrow transplant from a perfectly matched donor (122, 123). But, due to the recurrence of leukemia, this Berlin patient received another round of chemotherapy along with hematopoietic stem cell (HSC) transplant from the same donor. Despite the discontinuation of ART, no traces of viral load were found in the body since that time indicating that HIV-resistant T cells developed from donor CCR5-negative HSC were eventually able to suppress HIV replication (124).

Following this, about six more individuals were reported who went through similar process but complication in transplant and underlying cancer did not confer them a chance to survive and thus this Berlin patient documented as the first patient cured for the HIV infection (125). In 2016, at the Conference on Retroviruses and Opportunistic Infections, another case was reported which was similar to previously described ones. No signs of viral load were found after performing different assays; however, in this case, the ART has not interrupted yet. Therefore, considering this patient as another paradigm for the complete cure of HIV infection will be ahead of time (126).

## Gene Editing Tools for HIV Treatment

Initially in 1996, a genetic mutation was discovered in CCR5 which involves the deletion of 32 base pairs (from nucleotide 794 to 825) from normal protein (127, 128). Following this deletion, a frame shift mutation was created in the protein with the addition of seven unique amino acids which eventually generates a mutant allele having 215 amino acids. Evidences from different studies suggested that this genetic mutation proved to be a blessing as the individual homozygous for this mutation acquired protection against HIV (128, 129). By using this approach, Hütter et al. conducted a stem cell-based bone marrow transplant on HIV-infected patients. The individuals, homozygous for CR5delta-32(Δ32) mutation, were used as donor for this transplant. The results showed that after the transplant HIV-positive patients became HIV negative (130).

However, such bone marrow transplants have to face many complexities as the chance of finding a compatible donor homozygous for the mutation is very rare. Therefore, in order to cope with these challenges, scientists employed different gene editing strategies by using zinc finger nucleases (ZFN), Transcription activator-like effector nucleases (TALENs), and crisper cas 9 to target CCR5 for HIV treatment.

### Zinc Finger Nucleases

Zinc finger nuclease is a type of engineered protein having a zinc finger domain which is able to bind with specific regions of DNA and can perform the process of gene editing by producing a double stranded break in the desired DNA (131). Tebas with his coworkers conducted experimental trials to disrupt CCR5 in autologous CD4+ T-cells by using ZFN. The trial turned out to be successful as patients treated with modified CD4+ cells exhibited lower HIV level and acquired resistance against HIV. However, the decline in the HIV level was slower than normal implying that it can delay the disease progression but cannot be considered as permanent cure for infection (132).

Moreover, the treatment outcome depends upon the person’s genotype status. Homozygous CCR5 delta 32 (CCR5 delta32/delta32) individual was resistant to CCR5-tropic HIV infection due to the absence of functional receptors from the cell surface, and heterozygous carriers were slow in AIDS progression. A study was conducted in which some participants exhibited longest delay in the recurrence of HIV. Later on, it was identified that the delay in recurrence was due to the presence of heterozygous CCR5 mutation. Thus, for a successful clinical application a biallelic knock out is required in individuals having two non-mutated CCR5 genes (132).

### Transcription Activator-Like Effector Nucleases

Transcription activator-like effector nucleases, a highly versatile and efficient tool with lower cytotoxic effects than ZFN, were developed to control the HIV infection by disrupting CCR5 gene. These nucleases are synthesized by combining domain of a non-specific nuclease (*Fok*I) and customizable DNA-binding domain derived from TALE. These DNA binding domains contain highly conserved 33–35 amino acid repeats (133). Mock and his coworkers executed experimental trial to knock out the CCR5 gene using TALENs. The results were inspiring as the modified CCR5 T cells developed protection against R-5 tropic HIV (134). This technique produces lower off target effects and can recognize one nucleotide unlike ZNF, which identify three nucleotides per domain. The ability to identify one nucleotide per domain instead of three helps to increase the specificity of technique (135). However, a crucial limitation of this technique is that these TALENs can cause genomic instability by producing rearrangements in target cell. Therefore, their use in therapeutics demands more optimized strategies (136).

### CRISPR/CAS9

A prokaryotic-derived powerful genome-editing technique CRISPR-Cas9 (Clustered regularly interspersed palindromic repeats-CRISPR associated 9) involves two major biological components: Cas9 and engineered single-guided RNA. Cas9 is guided by sgRNA which helps in recognition of complementary target sequence flanked by PAM sequence and thus, it guide Cas9 for cleavage. Out of different types of CRISPR/Cas9, type II system is widely used because of involvement of reduced number of Cas enzymes (137). Multiple repeated sequences (~21–28 bp) in CRISPR loci interspersed by variable spacer sequence holding correspondence to sequence within foreign genetic elements (protospacer) and Cas9 genes that are placed alongside these loci. Non-coding tracr RNA triggers the processing of pre-crRNA to mature cr-RNA through ribonuclease RNase III and Cas9 protein usage. A 2–5 nucleotide PAM (Protospacer adjacent motif) occurs at 3 termini of target sequence and facilitates in cleavage by Cas9. Cas9 has two domains, i.e., HNH and RuvC, for cleavage of complementary (target) sequence and non-complementary (non-target) strand, respectively. These double stranded breaks are then repaired by cellular repair pathways: either non-homologous ends joining (NHEJ) or homologous directed repair (137).

## Therapeutic Applications of CRISPR/CAS9 for HIV-1

Although antiretroviral therapy has been in use to keep HIV-1 replication restricted, due to virus’ ability to hide itself in other parts of the patient’s genome, it can reemerge later, once medication is stopped. Nevertheless, researchers have introduced the world with a powerful genome editing technique, CRISPR/Cas9, which can eliminate viral genome from infected person by manipulating and ultimately blocking HIV-1 provirus genome expression (138).

### CRISPR/Cas9-Mediated Strategies to Eradicate HIV-1

CRISPR/Cas9 can eradicate HIV through different strategies:

(1) Blockage of pre-integrated proviral dsDNA.
(2) Disruption of co-receptor CCR5.
(3) Latent provirus can be targeted for cleavage, i.e., excision of viral genome *via* specifically targeting LTR regions of HIV-1 and also distorting viral genes. Thus, it helps in viral genome assembly and budding during HIV-1 lifecycle.
(4) Last, CRISPR-mediated reactivation of latent provirus can aid in antiretroviral therapeutic removal of HIV infection.

The shortcoming of ART to eradicate latent viral reservoirs (pathogenic virus in dormant state) has led to the need for a powerful strategy to particularly target integrated HIV-1 proviral DNA. CRISPR/Cas9 is a therapeutic antiviral genome modulation tool. It has a role in excision and removal of integrated latent virus (139).

CRISPR/Cas9-mediated genome editing can reactivate latent provirus of HIV. CRISPR/Cas9 has the potential to do gene-specific transcriptional activation of hotspots which is present 200-bp upstream of TSS of HIV latent provirus. dCas9, a Cas9 mutant, which lacks nuclease activity, interacted with transcription activator domain VP64 to achieve gene expression editing and increased activation by using multiple sgRNAs. For further enhanced activation, a polypeptide scaffold Suntag is used by dCas9 that recruits multiple antibody-fusion proteins which helps in binding of dCas9 to multiple VP64. Selective binding of RNA aptamers to dimerized MS2 bacteriophage coat protein develops a synergistic activation mediator that mediates recruitment of multiple effectors domains to dcas9 complex. Thus, CRIPSR/Cas9 system provides possibility to target and induce transcription from latent HIV-1 provirus reservoirs (139). Targeted editing of CCR5 receptor *via* CRISPR/Cas9 can provide treatment to HIV-1. A rare homozygous 32-bp deletion in CCR5 gene (CCR5 delta 32) in individual makes it resistant to HIV-1 infection and, thus, prevents virus from entering T cells. A sterilizing cure for HIV is developed through transplantation of allogeneic donor (CCR5 delta32) HSPCs but removal of HIV from latent provirus reservoirs is yet to be disclosed. Treatment of HIV using allogeneic donors is not widely applicable because of rare occurrence of homozygous CCR5 delta 32 donors, immune suppression, bone marrow ablation, and difficulty in finding HLA-matched donors. Many genome editing techniques as ZFN and TALENs were performed to induce such beneficial modifications in CCR5 to eradicate HIV-1 infection but CRISPR/Cas9 has eclipsed all these because ZFN and TALENs requires much time and are expensive in doing target-specific genome engineering while CRISPR/Cas9 is relatively easy to use and needed a single-guided RNA to specify the target cleavage site and also provides on-target specificity. CRISPR/Cas9 just needed lentiviral vector having Cas9 and sgRNA to target CCR5 (139). CCR5 gene-specific knockout *via* CRISPR utilizing sgRNA and Cas9 transfection in HEK 293K, K562, and also in human CD34+ HSPC cells are achieved (140). This technology can bring deleterious variants of CCR5 that repeats naturally occurring CCR5 delta 32 and indicated permanent cellular immunization from infection and functional cure is real possibility (139).

### CRISPR/Cas9-Induced Viral Escape

Recently, researchers have found out that RNA-guided mutations *via* CRISPR system not only can inhibit HIV replication but, on the other hand, also provide resistance. Mutations can eradicate HIV infection but some makes the HIV more resistant on getting high rate of surveillance by new mutations so there developed a difficulty in therapeutic targeting *via* CRISPR. RNA sequence of escaped HIV at cas9 cleavage site is investigated, and analysis showed that it is not the viral RT, which is the cause of these mutations but it is one of the cellular repair mechanism, i.e., NHEJ which by producing indels has ability to impair DNA functionality. Cas9 become unable to identify the target sequence anymore. It does no harm to virus; instead enable it to replicate further (141).

RNA-guided Cas9 cleavage of DNA sequence through repair pathway NHEJ can bring indels (insertion–deletion) in sequence which suppresses viral gene replication. These indels were supposed to be lethal for virus but recently, it is discovered that it can produce replication competent viruses resistant to CRISPR/Cas9. Thus, this CRISPR-mediated therapy is on one hand contributing in HIV-1 inactivation and on the other side, accelerating a viral escape.

CRISPR, being DNA sequence-specific mutagen, gives an independent source of resistance mutation. Viral escape mechanism entails mutation in viral DNA sequence *via* sgRNA (141, 142). Single sgRNA can eliminate whole HIV-1 DNA by targeting two LTR at 5 and 3 prime ends of integrated HIV-1 DNA. SgRNA/Cas9 can inhibit latent viral DNA as well. Viral escape *via* CRISPR system occurred due to mutations in viral DNA sequence targeted by sgRNA. This was not much surprising keeping in mind the ability of HIV-1 to thrive in new mutations and build resistance to antiviral therapeutic drugs and human immune responsiveness. This resistance might be due to error-prone RT. Target sequence developed changes, unable to be identified and targeted by T cells with same machinery. Hence, resistance emerged to CRISPR/Cas9 tool (141). CRISPR/Cas9 has some safety issues as it shows off-target effects which could result in cellular transformation (139).

## Conclusion

Since the terrible outbreak of HIV, the world is still suffering from its deadly consequences. As HIV is derived from SIV, so many of the epidemiological, phylogenetic, and genomic characteristics of HIV are similar to those of SIV, and this strongly supports the idea of cross-species transmission. ART has significantly altered the HIV global epidemiology. Initially, antiviral drugs were administered as monotherapy, but later on, the concept of combination therapy was introduced that is known as HAART having the potential to reduce the mortality and morbidity related to HIV-1 infection. Although there is no absolute treatment for HIV, continuous effort of researchers for developing better therapeutic approaches has become fruitful with the advent of CRISPR/Cas9.

## Author Contributions

All authors listed, have made substantial, direct and intellectual contribution to the work, and approved it for publication.

## Conflict of Interest Statement

The authors declare that the research was conducted in the absence of any commercial or financial relationships that could be construed as a potential conflict of interest.
